# Differing Rates of Carbapenem Resistance in Gram-Negative Bacteria Within Lower and Higher Socioeconomic Classes in Eastern India

**DOI:** 10.7759/cureus.83661

**Published:** 2025-05-07

**Authors:** Debarghya Chakraborty, Triparna Roy, Priyankar Gangopadhyay

**Affiliations:** 1 Internal Medicine, Princess Royal University Hospital, London, GBR; 2 Biostatistics and Health Informatics, King's College London, London, GBR; 3 Microbiology, Institute of Post Graduate Medical Education & Research, Kolkata, IND

**Keywords:** : antibiotic stewardship, carbapenem-resistant, public health care, resistant gram-negative infection, socioeconomic disparities

## Abstract

Background

Over the last 50 years, medical science has witnessed the emergence of antibacterial resistance at a concerning rate. Southeast Asia has become a leading epicenter for the development and spread of carbapenem-resistant bacterial strains. Socioeconomic background, being a major health determinant, can heavily affect this rate of antibiotic resistance.

Methods

This retrospective study was conducted at a 1,700-bed tertiary care facility in Kolkata, India, between October 2023 and January 2024. A total of 248 culture results from urine, sputum, blood, wound, and ascitic fluid, which showed growth of Gram-negative bacilli (GNB) and coccobacilli (GNCB) during the study period, were analyzed. Medical records were reviewed to collect demographic and clinical data. The comparison was made between two population groups based on economic status, identified by ration cards as defined by the Government of West Bengal, India.

Results

This study assessed the prevalence and antibiotic resistance patterns of Gram-negative bacteria in clinical samples, focusing on urine, blood, sputum, wound, and ascitic fluid. The analysis revealed that GNB were widely distributed across sample types, while GNCB showed higher prevalence in blood and ascitic fluid. Antibiotic resistance was notably prevalent among GNB, with 49 (27.5%) resistant to imipenem and 50 (28.1%) to meropenem. GNCB exhibited lower resistance, with six (14.3%) resistant to imipenem, eight (19.0%) to meropenem, and seven (16.7%) to doripenem. A comparison of carbapenem resistance (CR) between higher and lower socioeconomic classes showed no statistically significant differences, with resistance rates of 15 (41.7%) and 96 (45.3%), respectively. Further analysis of the most common isolates (*Acinetobacter baumannii*, *Escherichia coli (E. coli)*, *Klebsiella pneumoniae*, and *Pseudomonas aeruginosa*) also revealed no significant correlation between socioeconomic status and resistance, emphasizing that antibiotic resistance is a widespread issue transcending economic boundaries.

Conclusion

This study examines CR in Gram-negative bacteria across socioeconomic groups in Eastern India, finding no significant differences in resistance between higher and lower economic classes. Both groups showed similar levels of resistance, highlighting that antibiotic misuse, overprescription, and inadequate stewardship are pervasive issues transcending economic boundaries. The study emphasizes the need for enhanced healthcare provider training, public education on antibiotic misuse, and policy interventions to improve sanitation and waste management. Effective antibiotic stewardship and public health measures are crucial for combating resistance and ensuring equitable health outcomes.

## Introduction

Over the last 50 years, medical science has witnessed the emergence of antibacterial resistance at a concerning rate. Southeast Asia has become a leading epicentre for the development and spread of resistant bacterial strains. The Global Research on Antimicrobial Resistance (GRAM) study in 2019 demonstrated that around one million deaths were associated with antibacterial resistance within South and Southeast Asia [1]. Within this region, India, the most populous country in Southeast Asia, has recorded the highest incidence of antibacterial drug resistance between 2017 and 2020 [2].

In light of increasing antibacterial resistance worldwide, antibacterial therapies from the carbapenem group have played a major role as a last resort beta-lactam antibacterial in treating severe Gram-negative bacilli (GNB) and coccobacilli bacterial infections. A few examples, namely, imipenem, meropenem, doripenem, ertapenem, panipenem, and biapenem, collectively known as carbapenems, belong to the Watch Group of essential medicines listed by the World Health Organization (WHO) [3].

However, within the last decade, the increasing rate of carbapenem resistance (CR), with the majority of cases occurring in Gram-negative bacteria, either coccobacilli or *Enterobacteriaceae bacilli*, has raised significant concern within the medical fraternity [4]. In India, researchers reported a high prevalence of CR in *Acinetobacter baumannii *(>70%), followed by *Klebsiella pneumoniae* (>50%), *Pseudomonas aeruginosa* (>40%), and *Escherichia coli *(*E. coli*) (>10%) [5]. 

The growing incidence of resistant bacterial strains is primarily driven by resistance genes, which are often carried on plasmids, making them easily transmissible. The primary mechanism of CR involves the breakdown of carbapenems by carbapenemase enzymes [6]. The Indian subcontinent has witnessed the highest number of cases of carbapenamase, like the New Delhi metallo-beta-lactamase (NDM) and OXO-48-producing carbapenem-resistant bacteria [6,7]. Nonenzymatic mechanisms of CR also include the loss of nonspecific porin channels in the outer membrane of Gram-negative bacteria. These porins facilitate the passive transport of hydrophilic small molecules, nutrients, and antibiotics across the otherwise impermeable membrane [8]. This occurs by the process of deleterious or defective mutations of porin-encoding genes, such as in porin Ompk-35 [8]. Similarly, overexpression of genes encoding efflux pumps such as MexAB-OprM can lead to increased movement of antibiotic molecules out of the bacterial cell, culminating in resistance [9,10]. The combination of porin loss and efflux pump overexpression in carbapenem-resistant bacteria may also lead to cross-resistance with other β-lactams and various antibiotic classes. This phenomenon is frequently observed in *Acinetobacter baumannii, *contributing to the growing risk of developing multidrug-resistant bacterial strains [11]. CR may also result from mutations or modifications that affect the production levels or binding affinity of penicillin-binding proteins. However, these mechanisms are rarely observed in *E. coli*, *Pseudomonas aeruginosa*, and *Acinetobacter baumannii *[12]. 

Socioeconomic background, being a major health determinant, can heavily affect this rate of antibiotic resistance. Lack of access to clean water, inappropriate hygiene habits, and poor sanitation due to a deprived socioeconomic background increase the transmissibility of the resistant genes, eventually contributing to antibiotic resistance [13]. Also, lack of access to formal healthcare services and inappropriate antibiotic stewardship practiced by prescribers can increase the spread of resistant strains, potentially creating a serious threat to public health [13,14]. This is how poor socioeconomic status can become a serious threat to the increasing rate of antibiotic resistance. 

Thus, this study aims to compare CR rates in patients between high and low socioeconomic populations in a tertiary hospital in Kolkata, Eastern India, which attracts patients from all over Eastern India. This study further observes whether CR differs between the two socioeconomic classes for the four most common resistant bacterial groups: *Acinetobacter baumannii*, *Klebsiella pneumoniae*, *Pseudomonas aeruginosa*, and *E. coli*. This will aid in understanding the differing rates of antibiotic resistance across populations from various socioeconomic classes and could guide more targeted public health policies. Such policies would help ensure improved antibiotic efficacy for all patient groups, addressing disparities in treatment outcomes.

## Materials and methods

Ethical considerations

The eligible patients were informed that routine cultures would be used for research retrospectively, with no change to their treatment plan. Written informed consent was obtained from every patient as a standard operating procedure of the department. Ethics Committee approval was not sought because the study was retrospective and observational. The Institutional Ethics Committee, as a matter of principle, waives the requirement of permission for investigator-initiated retrospective data analysis-driven projects. This exemption is in accordance with the institution’s policy, which states that retrospective studies using anonymised data and involving no deviation from standard care do not require formal review. Confidentiality and anonymity were meticulously maintained. All the tenets of the Helsinki Declaration relating to bioethics policy were strictly adhered to.

Materials and methods

This retrospective study was conducted at a 1,700-bed tertiary care facility in Kolkata, India, between October 2023 and January 2024. 248 culture results from urine, sputum, blood, wound, and ascitic fluid were identified that showed growth of GNB and coccobacilli during the study period. Only the first bacteremic episode for each patient presenting with sepsis was included in the analysis. Blood culture results acquired before the initiation of the antibiotic course were only included. No patients with a history of immunodeficiency, i.e., HIV infection, receiving chemotherapy were included. Cultures were performed by inoculation on cystine lactose electrolyte-deficient agar (urine), while all other samples were inoculated on both MacConkey agar and blood agar to test for the presence of pathogenic organisms, and incubated at 37°C for 24 hours. Preparation of the agar plate was done following existing literature [15]. Species identification was carried out manually by Gram-staining and biochemical tests (alkaline peptone water (APW), citrate, triple sugar iron (TSI), and urease). The details of these biochemical tests to identify bacterial species are explained in Table 1.

**Table 1:** Biochemical tests for the identification of Gram-negative bacteria +: positive finding; -: negative finding; K/A: alkaline top/acidic butt; A/A: acidic top/acidic butt; d: definite growth without pH change; G: gas production; H2S: hydrogen sulphide precipitate This table presents the results of biochemical tests used for the identification of various Gram-negative bacteria isolated from clinical samples. The organisms tested include *Escherichia coli*, *Klebsiella pneumoniae*, *Acinetobacter baumannii*, *Staphylococcus aureus*, *Enterobacter cloacae*, *Proteus mirabilis*, and *Pseudomonas aeruginosa*. The biochemical tests conducted are indole production, citrate utilisation, triple sugar iron (TSI) agar reactions, urease activity, catalase activity, coagulase activity, oxidase activity, and motility

Organism	Biochemical tests							
	Indole	Citrate	TSI	Urease	Catalase	Coagulase	Oxidase	Motility
Escherichia coli	+	-	K/A, G	-				
Klebsiella pneumoniae	+	+	A/A, G	+				
Acinetobacter baumannii	-	+		-	+	-	-	-
Staphylococcus aureus	-	+		+	+	+	-	-
Enterobacter cloacae	-	+	K/A, G	d				
Proteus mirabilis	-	d	K/A, H2S	+				
Pseudomonas aeruginosa	-	+		-	+	-	+	+

This was confirmed with an automated identification system, VITEK-2 (Biomerieux, Marcy-l'Étoile, France). Then, antibiotic susceptibility was performed using Kirby-Bauer disc diffusion interpretive criteria as per revised Clinical and Laboratory Standards Institute performance standards and confirmed using the automated antibiotic susceptibility test, VITEK-2 (Biomerieux). A total of 10 μg doses of each antibiotic in the carbapenem group (imipenem, meropenem, doripenem, ertapenem) were used to calculate sensitivity. Isolates were classified as resistant, intermediate, and sensitive to antibiotics based on the size of the zone of inhibition according to the Clinical Laboratory Standard Institute (CLSI) guidelines [16].

Microbial resistance data have been extracted from de-identified patient reports, after regular tests and cultures as part of ongoing investigations and treatment plans. Demographic data were matched with these samples from a de-identified dataset. The comparison was made between two population groups based on economic status. This was identified by food subsidy cards or ration cards (AAY/PHH, lower socioeconomic class; GEN, higher socioeconomic class) as defined by the Government of West Bengal, India [17]. The population with income below the poverty line is classified as either AAY or PHH, while the population with income above the poverty line is classified as GEN.

Statistical analysis

Statistical analysis was performed to compare the antibiotic resistance patterns between the two socioeconomic classes. The resistance patterns were expressed as categorical outcomes (resistant or sensitive (including both intermediate and sensitive groups)) for each antibiotic. A Chi-square test of independence was utilised to understand the association between socioeconomic status and antibiotic resistance. A Chi-square test of independence was chosen to assess associations between categorical variables, as it is a robust and widely accepted method for larger sample sizes (n = 248) and does not require assumptions of normal distribution, making it appropriate for evaluating frequency data. The analysis was conducted in R (version 4.4.2 (October 2024)). All analyses were conducted using reproducible coding practices, and outputs, including contingency tables and test statistics, were stored for reference.

## Results

Distribution of Gram-negative bacteria

**Figure 1:**
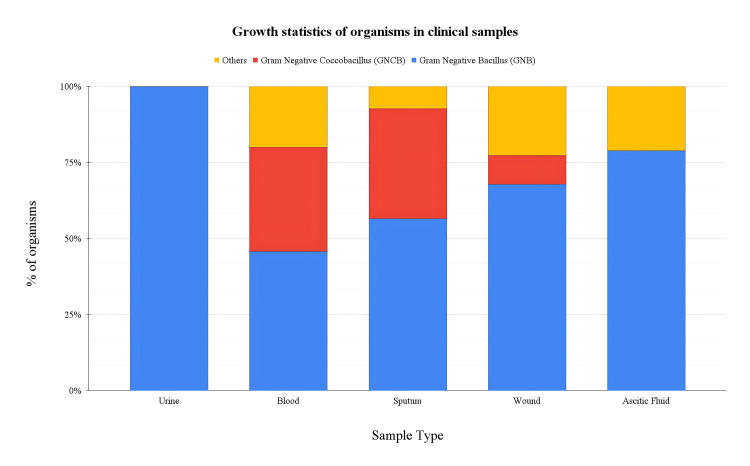
Growth statistics of organisms in clinical samples The bar graph depicts the distribution of Gram-negative coccobacilli (GNCB) and Gram-negative bacilli (GNB) across different clinical sample types: urine, blood, sputum, wound, and ascitic fluid

To assess the prevalence of Gram-negative bacteria within various clinical samples, we categorised the growth statistics for urine, blood, sputum, wound, and ascitic fluid samples. This classification provides a comprehensive overview of the bacterial distribution across different sample types, laying the groundwork for subsequent analysis of antibiotic resistance patterns (Figure 1).

Antibiotic resistance profile of GNB

**Figure 2:**
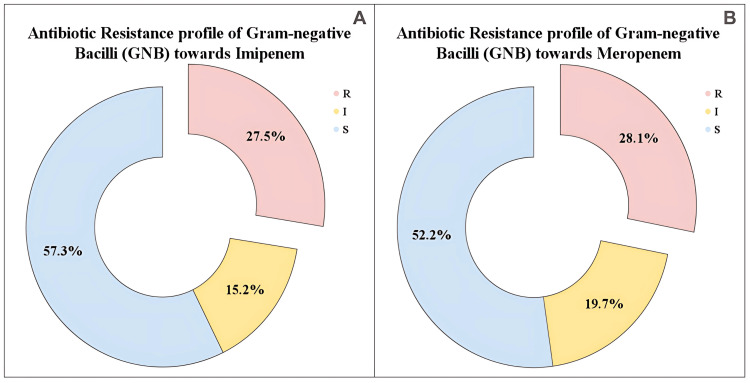
Antibiotic resistance profile of Gram-negative bacilli (GNB) R: resistant; I: intermediate; S: sensitive (A) Antibiotic resistance profile of Gram-negative bacilli towards imipenem and (B) antibiotic resistance profile of Gram-negative bacilli towards meropenem, respectively

To understand the antibiotic resistance profile of GNB, we analysed their resistance to imipenem and meropenem. The pie charts display the antibiotic resistance profile of GNB towards imipenem and meropenem. For imipenem, 49 (27.5%) of GNB isolates were resistant, 27 (15.2%) showed intermediate, and 102 (57.3%) were sensitive (Figure 2A). For meropenem, 50 (28.1%) of isolates were resistant, 35 (19.7%) were intermediate, and 93 (52.2%) were sensitive (Figure 2B). 

Antibiotic resistance profile of GNCB

**Figure 3:**
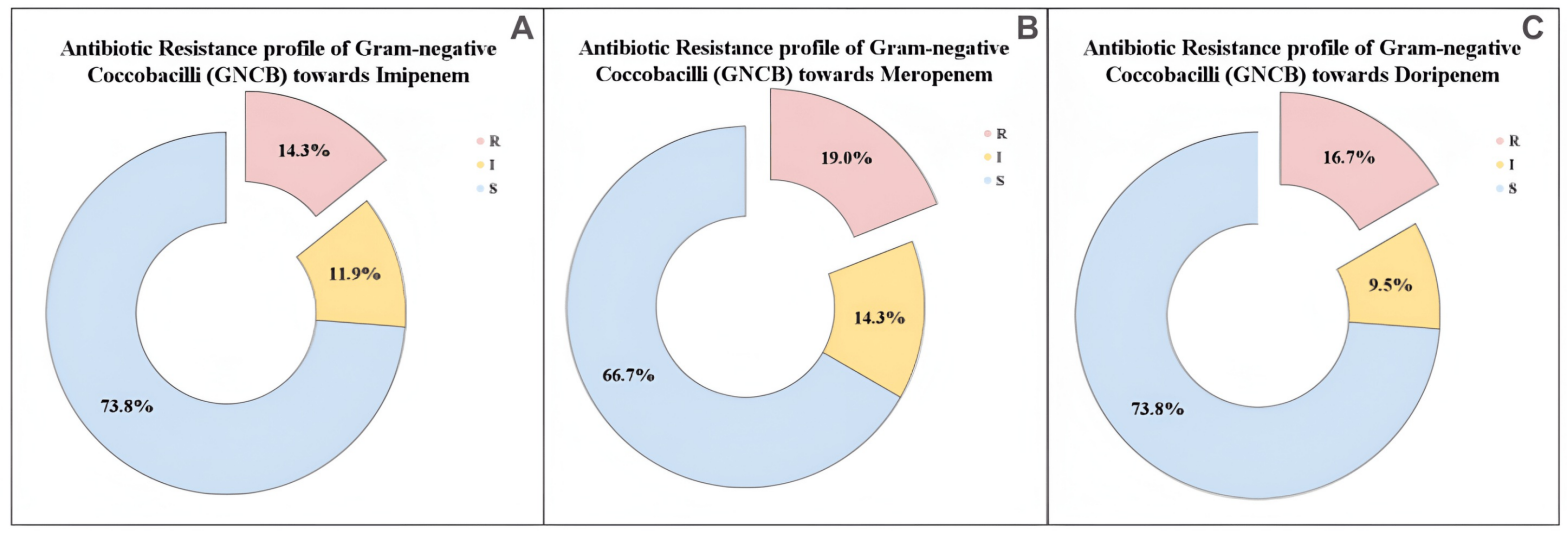
Antibiotic resistance profile of Gram-negative coccobacilli (GNCB) R: resistant; I: intermediate; S: sensitive (A) Antibiotic resistance profile of Gram-negative coccobacilli towards imipenem, (B) antibiotic resistance profile of Gram-negative coccobacilli towards meropenem, and (C) antibiotic resistance profile of Gram-negative coccobacilli towards doripenem, respectively

We further examined the antibiotic resistance profile of GNCB towards various carbapenem antibiotics. The pie charts illustrate the antibiotic resistance profile of GNCB towards imipenem, meropenem, and doripenem. For imipenem, six (14.3%) of GNCB isolates were resistant, five (11.9%) were intermediate, and 31 (73.8%) were sensitive (Figure 3A). For meropenem, eight (19.0%) were resistant, six (14.3%) were intermediate, and 28 (66.7%) were sensitive (Figure 3B). For doripenem, seven (16.7%) were resistant, four (9.5%) were intermediate, and 31 (73.8%) were sensitive (Figure 3C).

Socioeconomic status and CR

**Figure 4:**
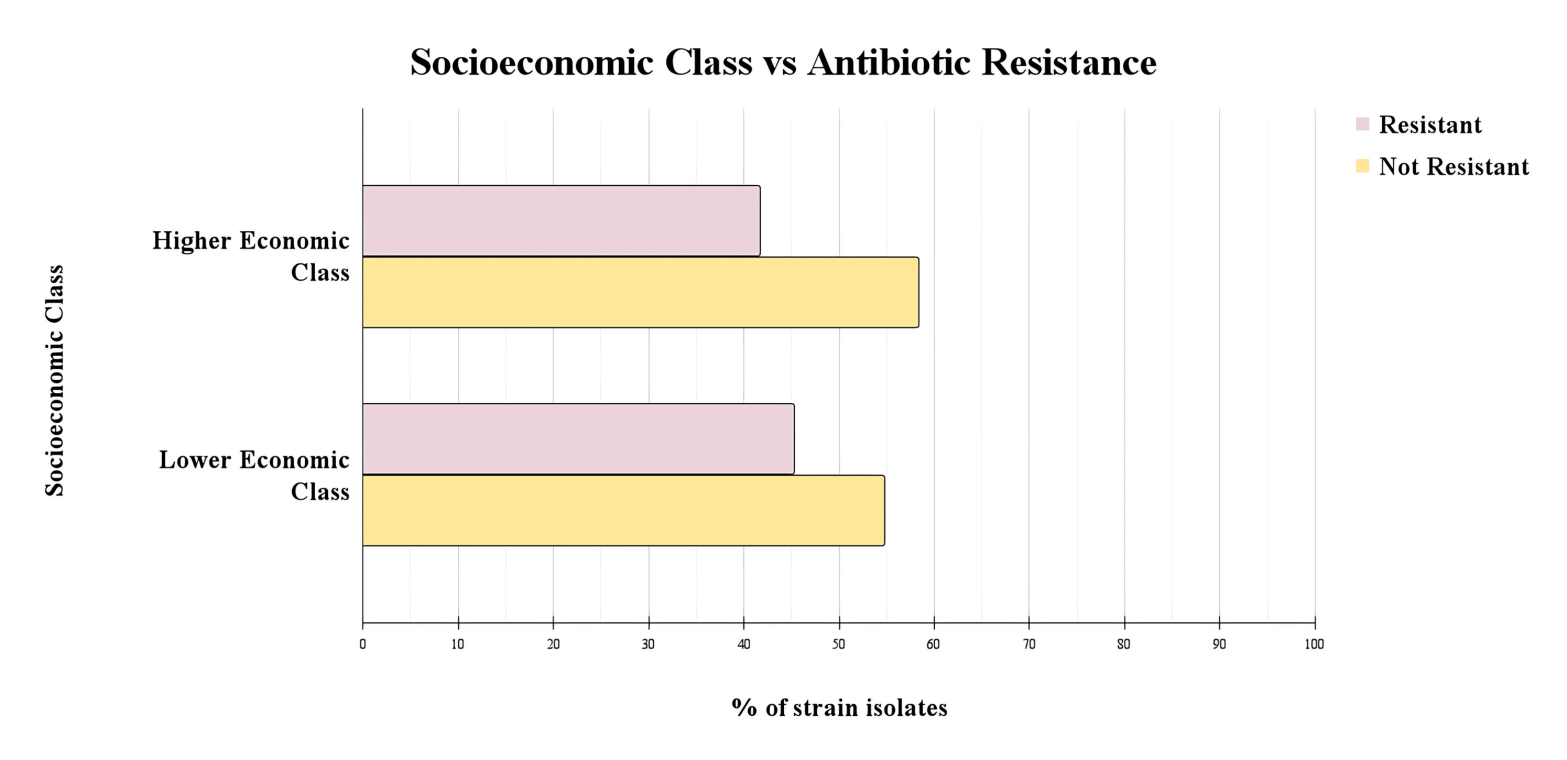
Socioeconomic class and carbapenem resistance This graph analysed whether carbapenem resistance varies with higher and lower socioeconomic classes. Statistical analysis revealed no significant association between socioeconomic class and carbapenem resistance (χ² (1, *N *= 248) = 0.163, *p* = .69), Fisher's exact test confirmed these findings (*p* = .721). The calculated odds ratio was 1.16 (95% CI: 0.54-2.56)

A total of 248 samples were analysed, categorised by socioeconomic class and resistance to carbapenems. Among the higher socioeconomic class, 15 (41.7%) were resistant, and 21 (58.3%) were not resistant (Figure 4). In the lower socioeconomic class, 96 (45.3%) were resistant, while 116 (54.7%) were not resistant (Figure 4). The analysis revealed no statistically significant association between socioeconomic class and CR (χ² (1, *N *= 248) = 0.163, *p* = .69).

**Figure 5:**
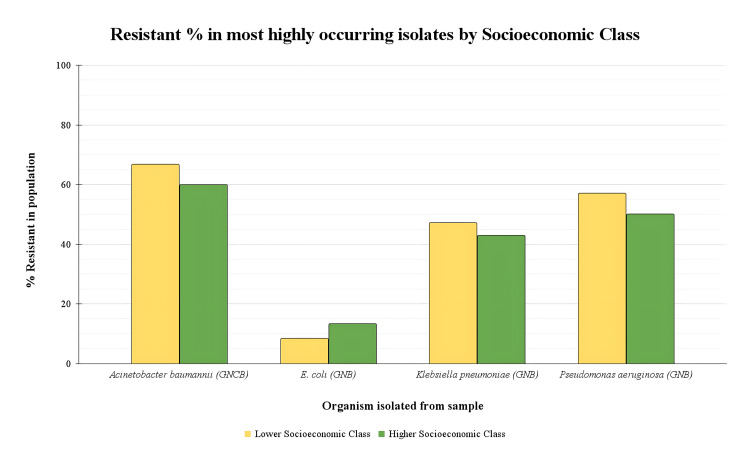
Socioeconomic class and comparison of resistant percentages in most prevalent bacterial isolate This graph analysed whether resistance percentages of *Acinetobacter baumannii*,*E. coli*, *Klebsiella pneumoniae*, and *Pseudomonas aeruginosa* differ between higher and lower socioeconomic classes. No statistically significant association was found between the socioeconomic class of patients and carbapenem resistance in any of the bacterial species studied, namely, *Acinetobacter baumannii* (χ² (1,  N = 17) = 1.06, p = .304), *E. coli* (χ² (1, N = 158) = 1.33, p = .249), *Klebsiella pneumoniae* (χ² (1, N = 60) = 0.32, p = .57), and *Pseudomonas aeruginosa* (χ² (1, N = 11) = 0.985, p = .321)

Next, the samples were analysed to study the resistant percentage in the most highly occurring isolates by socioeconomic class to understand how specific organisms' resistance patterns differ across higher and lower socioeconomic classes. The resistant percentages of the four most prevalent bacterial strains were compared, namely, *Acinetobacter baumannii*, *E. coli*, *Klebsiella pneumoniae,* and *Pseudomonas aeruginosa* (Figure 5). Our findings indicate that there is no statistically significant relationship between socioeconomic class and CR in Gram-negative bacteria in the patient population studied, namely, *Acinetobacter baumannii *(χ² (1,* *N = 17) = 1.06,p = .304), *E. coli *(χ² (1, N= 158) = 1.33,p= .249), *Klebsiella pneumoniae* (χ² (1, N = 60) = 0.32,p= .57), and *Pseudomonas aeruginosa* (χ² (1, N = 11) = 0.985, p = .321).

## Discussion

This study aimed to identify the rate of CR in patients in a tertiary hospital in Eastern India. Further, in this study, the rate of CR among higher and lower socioeconomic classes was compared. In the first set of results, the rate of GNB and GNCB colonisation within the urine, blood, sputum, wound, and ascitic fluid samples was identified. These samples were then tested for resistance to different antibiotic agents within the carbapenem group. This showed high rates of CR up to 28% for GNB in the culture samples. The results are equivalent to recent studies in India, indicating increasing CR in the population compared to the rate of global CR. In 2019, a study of 8787 *Enterobacteriaceae *showed a resistance rate of carbapenem-resistant *Enterobacteriaceae *(CRE) of 4.5% across 64 medical centres across the globe [18], whereas different regions of India have recorded as high as 31.8% prevalence rate of CRE [19]. Compared to the GNB population, GNCB bacteria comprising a major population from *Acinetobacter baumannii* record much higher rates of CR up to around 75%, similar to multiple population studies in India [20]. 

Lack of antibiotic stewardship in the use of carbapenem drugs has been a major contributing factor in this high antibiotic resistance rate. Prescription of meropenem has shown a steep increase of 3.5 times from 2010 to 2014 [21]. Not just overuse of the carbapenem group of medications but also excessive prescription of the penem group of medications has increased the risk of CR. Penem is a unique beta-lactam antibiotic developed as a hybrid, combining structural elements from both penam (penicillin) and cepham (cephalosporin) nuclei [22]. These agents have a structural similarity with carbapenem agents. Also, compared to carbapenems, which are available as injectables, these medications from the penem group are much easier to dispense due to their availability as oral agents. A recent study showed that faropenem consumption increased from 7.4 million standard units to 18.9 million standard units within five years after its approval in 2010 (154% increase). Although meropenem use also rose during this period, the consumption of faropenem surpassed all carbapenems combined, namely, meropenem, imipenem, doripenem, and ertapenem, between 2010 to 2014 in India, which is concerning due to the risk of cross-resistance with carbapenems [21]. The incidence of such species as *E. coli *isolates carrying CTX-M-15-type extended-spectrum beta-lactamase (ESBL) enzymes, cross-resistant to carbapenem due to induced resistance to faropenem, has already been reported [23]. This demonstrates the overall gravity of this ongoing public health crisis. 

Not just a lack of antibiotic stewardship but also inadequate sanitation and the lack of proper pharmaceutical waste disposal policies have contributed to the rising prevalence of antibiotic resistance, which represents a significant threat to public health [14]. Economic status is a key health determinant both in India and globally. The poorest fifth of households have a life expectancy of 65.1 years, compared to 72.7 years for the wealthiest fifth of households [24]. Thus, this study tried to answer whether antibiotic resistance rates differ between the two socioeconomic groups. 

Interestingly, however, no significant difference in the rate of antibiotic resistance among the two economic groups was observed. This study contradicts existing literature. A study focusing on South India showed that women in the lower 50th percentile of the income distribution had a higher likelihood of experiencing bacteriuria and were significantly more likely to have bacteriuria caused by ESBL-producing organisms [25]. This was attributed to a lack of availability of formal healthcare, including doctors for economic groups, and thus increased reliance on treatment prescriptions from informal healthcare providers. 

This disparity of results compared to existing literature can be explained by a study conducted in 2019 in Eastern India focusing on increasing antibiotic prescriptions. Despite higher theoretical knowledge regarding antibiotic prescription and resistance in doctors compared to informal healthcare providers like pharmacists, minimal difference was noticed in practice. For the common cold or sore throat, a similar percentage of physicians (88%) and informal healthcare providers (85%) considered antibiotic prescription [26]. Also, pressure from patients and pharmaceutical company representatives impacted the antibiotic prescription for physicians. This is further supported by another study, which failed to show any better antibiotic stewardship in doctors compared to informal healthcare providers. Interestingly, this study also highlighted better antibiotic prescription attitudes among medical students than among doctors [27]. This highlights the need for continuing training for healthcare professionals to ensure better antibiotic stewardship. It was also noted that while doctors recognised the importance of completing the full course of antibiotics to prevent resistance, many felt that in practice, shorter antibiotic courses were often necessary to assess patient response more effectively. A majority of doctors admitted to prescribing antibiotics without performing culture or sensitivity tests. Additionally, doctors often prescribe antibiotics as a precaution against secondary infections, a practice influenced by factors such as insufficient follow-up and diagnostic uncertainty [28].

Despite the influence of economic background on a patient’s choice to seek healthcare from either formal physicians or informal healthcare providers, the lack of effective antibiotic stewardship across both sectors exacerbates the risk of antibiotic resistance, regardless of the patient’s financial status. Our findings highlight that there are no significant differences in CR between high and low socioeconomic groups, underlining that antibiotic misuse is a widespread issue that cuts across socioeconomic lines. The root of this issue lies not only in the overprescription or inappropriate use of antibiotics but also in the lack of adherence to guidelines by both formal healthcare providers, such as physicians, and informal providers, who may not have access to the latest medical knowledge or resources.

Another contributing factor to the observed results could be due to the patient's choice of a tertiary government vs a private hospital. A recent study has shown that the population of lower socioeconomic backgrounds preferred government tertiary hospitals, whereas the majority of the higher socioeconomic background population opted for private tertiary medical care in Kolkata [29]. As a result, comparing high and low socioeconomic background populations in tertiary government hospitals can skew the results towards the majority, resulting in observed nonsignificant differences in antibiotic resistance. Thus, to further strengthen our findings, we suggest comparing the rate of CR in lower socioeconomic groups in government hospitals to higher socioeconomic groups in private hospitals.

To reduce confounders, patients with no background of immunodeficiency were recruited as they are more prone to have resistant infections. Similarly, this patient group was avoided as repeated infection and antibiotic use could have also contributed to heightened resistance risks in them. Yet, the results observed in this study can be hindered due to multiple limitations. Age and sex can affect the resistance rate. A study showed that men have a higher prevalence of developing antibiotic resistance. It also highlighted that different microbial resistance rates peak at different ages of the population [30]. In our study, no comparison was drawn for variation of resistance rate according to age and sex. Additionally, patients with a previous history of invasive procedures or long hospital stays can also increase the chances of antibiotic resistance, which contributes to major confounding factors in the study. Also, methods limitations can affect the observed results as follows. The sample size for certain bacterial isolates, such as *Acinetobacter baumannii* (n = 17), was small, reducing the statistical power to detect meaningful differences in resistance patterns within these subgroups. As a result, the findings related to these isolates should be interpreted with caution. Second, the retrospective and observational nature of the study, while suitable for describing resistance trends, limits the ability to draw causal inferences, as the data are collected from existing records, without randomisation or experimental control. This design makes it difficult to establish temporal relationships, whether an exposure preceded an outcome, and increases the risk of confounding, as important variables (e.g., comorbidities, prior antibiotic use) may not have been recorded or adjusted for. These limitations highlight the need for future prospective studies with larger, more evenly distributed sample sizes and multivariable analyses to better understand the determinants of antimicrobial resistance across different socioeconomic contexts.

Despite limitations, this study highlights key gaps in healthcare practices that can contribute to increasing antibiotic resistance. To address the growing public health threat posed by antibiotic resistance, including CR, it is essential to implement a multifaceted approach. This should involve increasing knowledge and providing training to informal healthcare providers, ensuring that they understand the importance of appropriate antibiotic use. Additionally, continued education for formal healthcare providers should be prioritised to reinforce best practices in antibiotic stewardship. Public awareness campaigns are also crucial to inform the general population about the risks of antibiotic misuse and the long-term consequences of antibiotic resistance. Only through a coordinated effort across all sectors can we mitigate this major public health crisis.

## Conclusions

This study investigates CR rates in Gram-negative bacteria among different socioeconomic classes in Eastern India. Despite initial hypotheses suggesting that socioeconomic class might influence resistance rates, our findings reveal no significant differences between higher and lower socioeconomic classes. This indicates that the challenge of antibiotic resistance transcends economic boundaries. The results underscore the pervasive issue of antibiotic misuse and overprescription, prevalent across both formal healthcare systems and informal providers. Additionally, improper sanitation and pharmaceutical waste disposal further contribute to the rise in resistant bacterial strains. These findings highlight the need for comprehensive public health strategies that include enhanced training for both formal and informal healthcare providers, emphasising the importance of appropriate antibiotic use across all socioeconomic classes. However, limitations such as the small sample size for certain isolates, unadjusted confounding variables like age, sex, and comorbidities, and the retrospective study design may have influenced the results and should be considered when interpreting these findings. Ultimately, addressing the growing public health threat of CR requires a coordinated effort to ensure improved antibiotic efficacy and equitable health outcomes for all populations.
